# INTEREST GROUP SESSION—EPIDEMIOLOGY OF AGING: BIOSOCIAL RESEARCH ON BRAIN AGING AND BIOLOGICAL AGING

**DOI:** 10.1093/geroni/igz038.1259

**Published:** 2019-11-08

**Authors:** Daniel W Belsky

**Affiliations:** Columbia University Mailman School of Public Health, New York, New York, United States

## Abstract

Our aging global population presents a new set of challenges for public health. Individual-disease focused models are becoming outmoded as geriatricians recognize multimorbidity and frailty as the central challenges in preserving health for older adults. Evidence from research into the biology of aging suggests that a set of common cellular-level processes underpin decline in system integrity that induces vulnerability to disease across multiple organ systems, including the brain. In parallel, research in life-course gerontology indicates that the roots of aging-related decline in system integrity extend from early life and encompass histories of social, psychological, and biochemical exposures. The research presented in this symposium aims to integrate these emerging paradigms in aging research by mapping connections among measures of aging in the brain and body and social, psychological, and nutrition exposures. Our symposium focuses on (1) links between social-psychological determinants of health and biological aging in the brain and body; and (2) social and behavioral protective factors that may buffer emerging biological risk in aging. The overarching goal of this symposium is to introduce an approach to gerontology that integrates geroscience with life-course social and psychiatric epidemiology to advance understanding of cognitive aging and functional decline, and ultimately identify novel interventions to extend healthy lifespan.

